# “DOACs for Left Ventricular Thrombus: Persistent Equipoise Despite New Observational and Randomized Data”: comment

**DOI:** 10.1016/j.rpth.2025.103339

**Published:** 2025-12-29

**Authors:** Artur Dziewierz, Piotr Jarosz, Tomasz Rakowski

**Affiliations:** 1 Clinical Department of Cardiology and Cardiovascular Interventions, University Hospital, Krakow, Poland; 2 Second Department of Cardiology, Institute of Cardiology, Jagiellonian University Medical College, Krakow, Poland

We read with great interest the article by Elbenawi et al. [1], which compares the use of direct oral anticoagulants (DOACs) and vitamin K antagonists (VKAs) for the treatment of left ventricular thrombus (LVT). The authors are to be commended for assembling the largest single-center cohort to date on this clinically important topic. Their finding that DOACs offer efficacy similar to VKAs in preventing stroke and systemic embolism (SSE) and achieving thrombus resolution, but with a significantly lower risk of major bleeding, adds valuable data to a field characterized by uncertainty. However, we believe several limitations and the broader clinical context, including very recent randomized trial data, warrant further discussion [[2], [3], [4], [5], [6], [7], [8], [9], [10]].

The study’s retrospective design, as the authors acknowledge, is a key limitation. This design is susceptible to selection bias and unmeasured confounders. A critical imbalance between the groups was the rate of concurrent dual antiplatelet therapy, which was significantly higher in the VKA group (27% vs 13%) [1]. While the authors suggest this may explain the increased bleeding risk with VKAs, this baseline difference represents a major potential confounder for both bleeding and ischemic outcomes, which may not be fully mitigated by multivariable adjustment. Furthermore, the absence of data on time in the therapeutic range for the VKA group is a significant omission. Subtherapeutic international normalized ratios are a well-established cause of treatment failure with warfarin, and without data on time in the therapeutic range, it is difficult to ascertain whether the comparable efficacy of DOACs was demonstrated relative to optimally managed VKA therapy. The study's findings must also be viewed within the context of a conflicting and evolving body of evidence [[2], [3], [4], [5], [6], [7], [8], [9], [10]]. The influential retrospective study by Robinson et al. [2] reported a significantly higher risk of SSE with DOACs compared with warfarin. The recently published Rivaroxaban vs Warfarin in Acute Left Ventricular Thrombus Following Myocardial Infarction (RIVAWAR) trial by Shah et al. [3] – the largest randomized controlled trial on this topic – provides crucial, albeit complex, insights. The RIVAWAR trial found that rivaroxaban was noninferior to warfarin for LVT resolution. However, in contrast to the safety findings of Elbenawi et al. [1], the RIVAWAR trial reported numerically higher (though not statistically significant) rates of both ischemic stroke and major bleeding with rivaroxaban [3]. This discordance, particularly in bleeding outcomes, between a large retrospective cohort and a large randomized clinical trial highlights the persistent clinical equipoise and the potential for confounding in observational data (Table). While the 2022 American Heart Association Scientific Statement deemed DOACs a "reasonable alternative" to VKAs, it did so based on insufficiently powered randomized data, a gap that the RIVAWAR trial begins to fill, even though it raises new questions about safety [4]. Elbenawi et al. [1] introduce an intriguing hypothesis regarding the use of a loading dose for DOACs, noting that early embolic events in their DOAC cohort occurred exclusively in patients who did not receive a loading dose. This observation is particularly relevant, given that the highest risk of embolization occurs in the initial weeks following LVT diagnosis, and it warrants investigation in future prospective trials.

**Table:** Comparison of key outcomes in selected studies on anticoagulation for left ventricular thrombus.

Study	Design	*N*	SSE	Major bleeding
Elbenawi et al. [1]	Retrospective	182	DOAC 5.7% vs VKA 5.4% (*P* = 1.00)	DOAC 1.4% vs VKA 9.0% (*P* = .02)
Robinson et al. [2]	Retrospective	514	Higher risk with DOACs (adjusted HR, 2.64)	Not a primary outcome
Shah et al. [3]	Randomized clinical trial	261	Rivaroxaban 3.5% vs warfarin 1.1% (*P* = .43)	Rivaroxaban 2.3% vs warfarin 1.1% (*P* = .66)

DOAC, direct oral anticoagulant; HR, hazard ratio; SSE, stroke and systemic embolism; VKA, vitamin K antagonist.

In conclusion, the study by Elbenawi et al. [1] is an important contribution. However, when contextualized with conflicting observational data and the nuanced results of the RIVAWAR randomized clinical trail, it is clear that the optimal anticoagulant for LVT remains unsettled [[2], [3], [4], [5], [6], [7], [8], [9], [10]]. The question can only be definitively answered by a large, multicenter randomized clinical trial adequately powered to detect differences in clinical endpoints, such as SSE and major bleeding.
